# Hyaluronan Accumulates in Inflamed Lymph Nodes and Promotes B-Cell Activation

**DOI:** 10.1002/pgr2.70031

**Published:** 2025-08-05

**Authors:** Zhiwei Li, Nadine Nagy, Gernot Kaber, Payton L. Marshall, Naomi L. Haddock, Everett Meyer, Robert R. Vernon, Manish J. Butte, Ovijit Chaudhuri, Thomas N. Wight, Paul L. Bollyky

**Affiliations:** 1 Department of Medicine, Division of Infectious Diseases and Geographic Medicine, Stanford University, Stanford, California, USA; 2 Department of Medicine, Division of Blood and Marrow Transplantation & Cellular Therapy, Stanford University, Stanford, California, USA; 3 Department of Medicine, Division of Oncology, Stanford University, Stanford, California, USA; 4 Department of Medicine, Division of Blood and Marrow Transplantation, Stanford University, Stanford, California, USA; 5 Matrix Biology Program, Center for Fundamental Immunology, Benaroya Research Institute, Seattle, Washington, USA; 6 Departments of Pediatrics and Microbiology, Immunology, and Molecular Genetics, Division of Immunology, Allergy, and Rheumatology, University of California Los Angeles, Los Angeles, California, USA; 7 Department of Mechanical Engineering, Stanford University, Stanford, California, USA

**Keywords:** 4-methylumbelliferone, autoimmune diabetes, B cells, hyaluronan, lymph nodes

## Abstract

The extracellular matrix component hyaluronan (HA) plays important roles in inflammation and immune regulation. However, its involvement in lymphoid tissue during autoreactive processes remains poorly understood. Here, using the DORmO mouse model of autoimmune diabetes, we demonstrate that HA progressively accumulates in lymph nodes and splenic germinal centers during disease development. This accumulation is accompanied by a shift from high to low molecular mass HA fragments. Through immunofluorescence microscopy, we observed extensive colocalization between HA deposits and B cells in germinal centers. Inhibition of HA synthesis using 4-methylumbelliferone (4-MU) increased lymph node stiffness. Treatment with 4-MU decreased immunoglobulin production and reduced B cell activation and differentiation into plasmablasts following immunization. These findings reveal a previously unrecognized role for HA in regulating B cell responses within lymphoid tissues during autoimmune reactions and suggest that targeting HA synthesis may represent a novel therapeutic strategy for conditions involving dysregulated B cell responses.

## 1 | Introduction

The extracellular matrix (ECM) plays a fundamental role in regulating immune responses through both structural and signaling functions [1]. The ECM forms a dynamic and complex network of proteins and glycosaminoglycans, such as collagen and hyaluronan (HA), that provides not only structural support but also biochemical and mechanical signals essential for immune cell mobility, survival, and function [2]. These signals integrate with other factors, such as cytokines and mechanosensitive cues, to regulate immune cell positioning, activation, and interactions within the microenvironment [2].

Among ECM components, HA, a non-sulfated glycosaminoglycan, has emerged as a critical mediator of inflammation and immune cell activation [3]. In healthy tissues, HA exists predominantly in high molecular weight forms which help maintain tissue homeostasis [4–6]. However, during inflammation, HA is actively synthesized and processed into lower molecular weight fragments that can have distinct biological effects [7].

These HA fragments can serve as damage-associated molecular patterns (DAMPs) that trigger inflammatory responses and modulate immune cell function [7–11]. The production of HA is regulated by three HA synthases (HAS1–3) that catalyze HA synthesis from two activated sugar precursors, UDP-glucuronic acid (UDP-GlcUA) and UDP-N-acetyl-glucosamine (UDP-GlcNAc) [12]. While its degradation is controlled by hyaluronidases and reactive oxygen species [12].

Lymphoid organs like lymph nodes and the spleen are critical sites for antigen presentation and lymphocyte activation. The mechanical and chemical properties of the ECM have been shown to be crucial to these processes [13–15]. Recent studies have revealed that disease states can dramatically alter both the amount and size distribution of HA in affected tissues [11, 16–19]. Of particular interest is the accumulation of HA in lymphoid organs and islets during autoimmune diabetes development, where it may influence immune cell activation and fate decisions [17–19]. This accumulation is accompanied by a shift from high molecular weight HA to lower molecular weight fragments in the islets [11] and changes in HA-binding proteins [19]. These changes in HA content and size significantly impact T cell responses, including their trafficking, activation, and polarization [20, 21].

While T cells have been traditionally considered the primary mediators of β cell destruction in type 1 diabetes (T1D), growing evidence suggests that B cells play critical roles in disease pathogenesis, involving multiple mechanisms such as serving as efficient antigen-presenting cells for autoreactive T cells, producing autoantibodies against islet antigens, and secreting inflammatory cytokines [22]. Indeed, studies in nonobese diabetic (NOD) mice have demonstrated that B cell deficiency prevents diabetes development [23], and B cell depletion can even restore pancreatic β cell function [24]. While our understanding of B cell contributions to T1D has grown substantially, the role of the tissue microenvironment, particularly HA, in modulating B cell responses remains poorly understood. Our previous observations of altered HA deposition and fragmentation in lymphoid tissues during autoimmune responses raise important questions about how these changes might influence B cell activation and function [18, 19].

The DORmO mouse model of autoimmune diabetes provides an excellent system for investigating these questions, as it allows temporal analysis of HA accumulation and immune cell activation in both lymphoid tissues and target organs [17, 25]. These mice are generated by crossing DO11.10 mice, which carry a T cell receptor transgene specific for ovalbumin (OVA), with RIP-mOVA mice that express membrane-bound OVA on pancreatic β cells. This combination results in synchronized autoimmune responses against β cells, with insulitis beginning at 4 weeks of age and nearly all mice progressing to diabetes by 15 weeks [17, 25]. The predictable disease progression in this model enables detailed temporal analysis of HA accumulation and its relationship to immune activation.

4-methylumbelliferone (4-MU), a specific inhibitor of HA synthesis, offers a valuable tool for manipulating HA levels in vivo to assess its functional importance [26]. 4-MU acts through multiple mechanisms including depletion of UDP-glucuronic acid pools and reduction of HAS expression [26]. The effects of 4-MU on HA synthesis have been well-documented across multiple cell types and tissues, making it an ideal tool for investigating the role of HA in immune responses [26].

Here, using the DORmO mouse model and 4-MU, we examined how HA accumulation in lymphoid tissues influences B cell responses during immune processes. We investigated the temporal and spatial patterns of HA deposition in lymphoid tissues (lymph nodes and spleen) during autoimmune diabetes development and its relationship to B cell localization. Using immunization models, we further evaluated whether pharmacological inhibition of HA synthesis can modulate B cell activation and antibody production. Our findings provide new insights into how ECM remodeling in lymphoid tissues influences B cell function and suggest potential therapeutic strategies for conditions involving dysregulated B cell responses.

## 2 | Materials and Methods

### 2.1 | Mouse Models and Housing

DORmO double-transgenic mice were generated by crossing DO11.10 mice with BALB/c mice expressing RIPmOVA. DO11.10 transgenic mice were obtained from The Jackson Laboratory, while RIPmOVA transgenic mice were provided by Benaroya Research Institute [17]. Age and sex-matched BALB/c mice served as controls. To inhibit HA synthesis in vivo, mice were treated with 4-MU-supplemented chow prepared as previously described [11]. 4-MU (Alfa Aesar) was incorporated into standard mouse chow by TestDiet, followed by irradiation before delivery. This formulation has been previously shown to deliver 250 mg/mouse/day, achieving a plasma drug concentration of 640.3 ± 17.2 nmol/L as measured by HPLC-MS [27]. Treatment was initiated when mice reached 6 weeks of age and continued until the experimental endpoint.

For immunization studies, DO11.10 mice received the same 4-MU treatment regimen starting at 6 weeks of age. Mice were immunized with OVA in alum twice, separated by 14 days. 10 days after the second immunization, blood samples were collected for serum immunoglobulin analysis, and spleen and lymph nodes were harvested for flow cytometric analysis of immune cell populations. This protocol was as previously reported by us [18].

Mice were housed in Stanford University’s vivarium under specific pathogen-free conditions with unrestricted access to standard chow and water. All experimental procedures were conducted in accordance with protocols approved by Stanford University’s Institutional Animal Care and Use Committee.

### 2.2 | Disease Monitoring

Diabetes monitoring was performed as previously reported [17]. In brief, diabetes development was tracked by measuring blood glucose levels weekly starting at 4 weeks of age. Blood samples were collected through saphenous vein puncture and analyzed using a Contour blood glucose meter and blood glucose monitoring strips (Bayer Healthcare). Mice were classified as diabetic when blood glucose exceeded 250 mg/dL in two consecutive measurements.

### 2.3 | Tissue Analysis for HA

Following previously established protocols [17], we prepared tissue sections for HA visualization. Freshly harvested tissues underwent fixation in 10% neutral buffered formalin or methyl Carnoy’s solution, followed by paraffin embedding using an ASP300 processor (Leica Microsystems Inc.). We generated 5-μm-sections using a Leica RM 2255 microtome. To detect HA, we utilized biotinylated HA-binding protein (4 μg/mL in 0.1% BSA-PBS) in conjunction with a streptavidin-HRP detection system (Leica Microsystems Inc.). Microscopic evaluation was performed on a Leica DM IRB system, and images were captured using a Pursuit digital camera (Diagnostic Instruments). Tissue sections were then digitally scanned using a NanoZoomer system (Hamamatsu). Digital images were analyzed using Visiopharm software (Hoersholm) for automated quantification. The software was configured to distinguish HA-positive regions from background tissue based on pixel value thresholds. For lymph nodes, we measured both total node area and HA-positive regions, calculating the percentage of HA-positive area. In spleen sections, we quantified the number and size of white pulp regions and measured the proportion of HA-positive staining within these areas. To ensure consistency, all images were processed using identical threshold settings and analyzed in parallel. Areas were measured in square micrometers [19].

### 2.4 | HA Quantification

HA quantification using biochemistry approaches was performed as previously reported [11, 18]. For measuring tissue HA content, samples were first dehydrated through lyophilization and weighed. Dried tissues underwent overnight proteinase K digestion (250 μg/mL in ammonium acetate buffer, pH 7.0) at 60°C, followed by enzyme inactivation at 100°C for 20 min. HA levels were then determined using a competitive ELISA approach, where sample HA competed with plate-bound HA for binding to biotinylated HA-binding protein. The final signal inversely correlated with sample HA concentration.

### 2.5 | HA Molecular Size Distribution Analysis

The molecular size distribution of HA was determined using sequential ion exchange and size exclusion chromatography as previously described [11]. Tissue samples were processed through lyophilization, weighing, and proteolytic digestion using proteinase K (250 μg/mL in 100 mM ammonium acetate buffer, pH 7.0) at 60°C overnight, followed by enzyme inactivation at 100°C for 20 min. For ion exchange chromatography, we used DEAE-Sephacel columns (300 μL matrix bed prepared from 600 μL DEAE-Sephacel slurry) equilibrated with 8 M urea buffer. After sample application and washing with 10 mL of urea buffer, HA was eluted using 300 μL of urea buffer containing 0.25 M NaCl. For size separation, the DEAE-purified samples (200 μL containing 6–7 μg HA) were applied to a Sephacryl S-1000 column. Fractions (0.3 mL) were collected every 1.5 min, with vitamin B12 (10 μL) serving as an end marker. The column was eluted with PBS at a 12–15 mL/h flow rate. HA content in the collected fractions was quantified by ELISA to establish molecular size distribution patterns.

### 2.6 | Immunofluorescence Microscopy

Immunofluorescence staining and microscopy were performed as previously reported [21, 28]. In brief, for immunofluorescence analysis, deparaffinized tissue sections were blocked in PBS containing 2% normal goat serum. Sections were incubated with biotinylated HABP and anti-B220 antibody (RA3–6B2) overnight. For HA detection, Alexa-Fluor 488-streptavidin was used. B cells were visualized with Alexa-Fluor 546-conjugated goat anti-rat secondary antibody (Thermo Fisher), and nuclei were counterstained with DAPI. Images were acquired using a Leica TCS SP5 II scanning confocal microscope.

### 2.7 | Tissue Stiffness Measurements

Lymph node stiffness was analyzed by atomic force microscopy using an MFP-3D Bio system (Asylum Research) as previously described [11]. We used a silicon probe with a 5–10 μm colloidal borosilicate glass bead. For measurements, tissue samples were gently indented in contact mode by the cantilever tip with a trigger force of 500 pN. Deflection data were converted to force by multiplying by the spring constant. Young’s modulus was determined by analyzing the sample indentation curves during tip extension. We applied the Hertz model within the Asylum Research software to fit the force-indentation relationship. For tissue analysis, a Poisson’s value of 0.5 was applied along with standard default parameters. The resulting stiffness measurements underwent statistical analysis using R software, with significance determined through permutation testing [11].

### 2.8 | Flow Cytometry

Flow cytometry staining and analysis protocols were performed as previously reported by us [18]. In brief, cells were stained and analyzed on an LSR II flow cytometer (BD Biosciences). The following antibodies were used: CD19 (clone 6D5), CD69 (clone H1.2F3), B220 (RA3–6B2), and CD138 (clone 281–2) (BioLegend). Data analysis was performed using FlowJo v10 software.

### 2.9 | Statistical Analysis

Data are expressed as mean ± SEM of *n* independent measurements. Comparison between the two groups was performed with unpaired *t*-tests. A *p* < 0.05 was considered statistically significant. Data analysis was performed using GraphPad Prism software.

## 3 | Results

### 3.1 | HA Accumulates in Lymph Nodes During Autoimmune Diabetes Development

The DORmO mouse model develops insulitis by 4 weeks of age and progresses to hyperglycemia (blood glucose > 250 mg/dL) by 15 weeks (Figure 1A). We examined HA deposition in pancreatic lymph nodes (pLN). Immunohistochemistry revealed progressive accumulation of HA in pLN of DORmO mice compared to BALB/c controls (Figure 1B). Quantification showed significantly increased HA-positive area in DORmO pLN at 15 weeks compared to control animals (Figure 1C), correlating with disease progression and blood glucose elevation. Despite increased HA content, overall lymph node size remained unchanged between groups (Figure 1D), suggesting that HA accumulation reflects qualitative changes in the lymph node microenvironment rather than organ enlargement.

### 3.2 | Low Molecular Mass (LMM) HA Dominates in Inflamed Lymph Nodes

To characterize the molecular mass distribution of accumulated HA, we performed size exclusion chromatography on pLN extracts. At 4 weeks, before disease onset, DORmO mice showed predominantly high molecular mass (HMM)-HA, with peaks at lower Kav values (Figure 2A). As the disease progressed, DORmO pLN exhibited a gradual shift toward LMM-HA fragments, as indicated by peaks moving to higher Kav values at 10 and 15 weeks (Figure 2B,C). In contrast, control mice showed an opposite trend over time, with HA distribution progressively shifting toward HMM, as indicated by peaks moving to lower Kav values (Figure 2B,C). This size-distribution analysis reveals distinct patterns of HA processing between control and diseased lymph nodes during the course of the autoimmune response.

### 3.3 | HA Deposits Accumulate in Germinal Centers and Colocalize With B Cells

After examining HA accumulation in lymph nodes, we next investigated its distribution in the spleen, another major site of B cell responses. The white pulp of the spleen contains distinct B cell follicles, where germinal centers form during immune responses. Immunohistochemical staining revealed accumulation of HA specifically within these follicular regions of DORmO mice compared to control animals (Figure 3A). Total splenic HA content was significantly elevated in DORmO mice (Figure 3B). Further analysis of the white pulp regions showed significantly increased HA-positive area at 10 and 15 weeks (Figure 3C). While the number of white pulp regions remained constant between groups (Figure 3D), their size showed a trend toward increase in DORmO mice, though this did not reach statistical significance (Figure 3E), suggesting expansion of B cell zones during autoimmune response. The preferential accumulation of HA in these B cell-rich areas raised the possibility that HA might directly influence B cell responses within germinal centers, where B cells undergo activation, proliferation, and differentiation [29].

Using multi-color immunofluorescence microscopy, we observed extensive colocalization between HA deposits and B220 + B cells in DORmO mouse germinal centers (Figure 4A–C). HA was particularly abundant in the subcapsular sinus floor and sites of sinus invagination (Figure 4A), as well as throughout the lymphatic sinuses (Figure 4B). Further analysis revealed intimate contact between HA matrices and B cells within germinal centers (Figure 4C), suggesting potential direct interactions between HA and B cells during activation.

### 3.4 | 4-MU Treatment Alters Lymph Node Stiffness and Inhibits B Cell Responses

Since ECM composition can influence tissue mechanics and immune cell function, we investigated whether modulating HA synthesis affects lymph node physical properties. Treatment with 4-MU, an inhibitor of HA synthesis, significantly increased pLN stiffness in both control and DORmO mice as measured by atomic force microscopy (Figure 5A). This mechanical change occurred without affecting total lymphocyte numbers in lymph nodes (Figure 5B), indicating that the increased tissue stiffness was not due to altered cellularity.

To examine how HA inhibition affects humoral immunity, we performed an immunization study comparing normal chow versus mice pretreated with 4-MU. In this experiment, all mice received two OVA/alum immunizations separated by 14 days, followed by analysis 10 days after the second immunization (Figure 6A). In 4-MU-treated mice, we observed significantly reduced IgM and IgG levels following immunization (Figure 6B,C). Flow cytometric analysis of immune cell populations showed a significant decrease in T cell frequency, while the proportion of B cells remained unchanged in the spleen (Figure 6D). However, despite maintained B cell numbers, 4-MU treatment profoundly impacted B cell function. Flow cytometric analysis revealed significant changes in B cell activation and differentiation following 4-MU treatment. In the spleen, 4-MU treatment significantly reduced the frequency of activated B cells (CD19 + CD69+) (Figure 6E), and a similar trend was observed in lymph nodes (Figure 6G). The impact extended to plasma cell differentiation, with 4-MU treatment markedly decreasing plasmablasts (CD19+B220^low^CD138^hi^) in both spleen (Figure 6F) and lymph nodes (Figure 6H). These consistent reductions in both activated B cells and plasmablasts across different lymphoid tissues suggest that HA is required for proper B cell responses during immune challenge.

These results, together with our findings of HA accumulation in germinal centers and its impact on tissue mechanics, demonstrate a correlation between HA composition in lymphoid tissues and B cell responses during immune activation. The shift from HMM to LMM-HA in lymphoid tissues during autoimmune response, coupled with the effects of HA inhibition on B cell activation and differentiation, indicates that HA accumulation and processing may be a key regulator of humoral immunity in both physiological and pathological contexts.

## 4 | Discussion

In this study, we demonstrate that HA accumulates in lymph nodes and splenic germinal centers during autoimmune diabetes, and this accumulation promotes B-cell activation. Furthermore, we show that pharmacological inhibition of HA synthesis using 4-MU can modulate B-cell responses, suggesting a potential therapeutic approach for autoimmune diseases.

The accumulation of HA in lymphoid tissues during inflammation represents a significant alteration in the ECM that may influence immune responses through multiple mechanisms [18, 19]. HA accumulation has been reported in various inflammatory conditions and different organs. In type 2 diabetes (T2D), HA levels are increased systemically including in serum and skeletal muscle, independent of glycemic control [30]. Furthermore, HA accumulates in pancreatic islets during T2D and contributes to β-cell loss [31]. During islet transplantation, infiltrating T cells can produce HA, primarily through upregulation of HAS1 and HAS2, while HAS3 is downregulated [32]. This pattern differs from what we previously observed in autoimmune diabetes, where HAS3 was upregulated in inflamed islets [17]. During liver cirrhosis, HA accumulates in hepatic tissues and can serve as a biomarker of disease progression [33]. In autoimmune arthritis, increased HA deposition is observed in synovial tissues [34]. Our findings of HA accumulation specifically in lymphoid tissues during autoimmune responses share some similarities with these conditions, particularly the association with inflammation. However, unlike T2D, where HA increases systemically [30], T1D shows a different pattern of HA distribution, with significant accumulation observed in pancreatic islets and lymphoid tissues [17, 19].

Studies have revealed that HA content can significantly influence tissue mechanical properties [11, 35, 36]. In pancreatic islets, increased HA accumulation during inflammation was shown to reduce tissue stiffness [11]. Consistent with this, in our study, we observed that 4-MU treatment led to increased tissue stiffness in lymphoid organs. Tissue stiffness has been shown to influence immune cell behavior and function [37, 38]. Therefore, understanding how HA-mediated changes in tissue mechanics affect immune responses in lymphoid organs represents an interesting area for future investigation.

B cells contribute to the pathogenesis of T1D through multiple mechanisms, including secretion of autoantibodies targeting islet autoantigens, serving as antigen-presenting cells to CD4 + T cells, participating in coexistence with CD8 + T cells around the islets to regulate early pancreatic microenvironment, and differentiating into regulatory B cells (Bregs) to exert protective effects against T1D [22]. Our observation that HA accumulation coincides with B-cell activation in germinal centers suggests a previously unrecognized role for the ECM in regulating these processes. The timing and location of HA deposition, particularly its enrichment in germinal centers, imply that HA may directly influence B-cell responses during autoimmune reactions. This adds to our understanding of how the tissue microenvironment shapes immune responses and suggests new therapeutic opportunities. While follicular regions are predominantly composed of B cells, other immune cells such as T cells, macrophages, and dendritic cells are also present and may be influenced by HA accumulation. Our focus on B cell responses is supported by the functional studies demonstrating the role of HA in B cell activation and antibody production, though future investigations of HA effects on other immune cell types in these regions would be valuable.

The therapeutic potential of targeting HA synthesis in autoimmune diseases has been supported by multiple studies [20, 39, 40], but our findings provide new mechanistic insights into how 4-MU might exert its effects through modulation of B-cell responses. Previous studies using B cell-depleting therapies like rituximab have shown promise in treating autoimmune diseases, including T1D [23, 24, 41]. However, these effects are often temporary, with disease progression resuming once B cells repopulate [42]. Our results suggest that targeting the tissue microenvironment through HA inhibition might offer a complementary approach, potentially leading to more durable therapeutic effects by altering the fundamental mechanisms of B-cell activation.

While our initial observations of HA accumulation were made in the DORmO autoimmune model, we extended our investigation to validate the relationship between HA and B cell responses using a standard immunization protocol. Using 4-MU to inhibit HA synthesis, we demonstrated that HA is required for optimal B cell activation, plasmablast differentiation, and antibody production during immune responses. Together with our findings in the DORmO model, these results establish HA as an important regulator of B cell function in immune processes, suggesting that targeting HA synthesis could be a viable therapeutic strategy for conditions involving dysregulated B cell responses.

Furthermore, our findings have broader implications for understanding how ECM remodeling influences immune responses. The traditional view of ECM as merely providing structural support has evolved [43], and our study adds to growing evidence that ECM components actively participate in immune regulation [13, 15, 28, 44–59]. The accumulation of HA and its effects on B-cell activation highlight how matrix molecules can serve as context-dependent immune modulators [6]. This suggests that other ECM components might similarly influence immune responses in ways that remain to be discovered.

Several aspects of our findings warrant further investigation. While we have demonstrated a correlation between HA accumulation and B-cell activation, the precise molecular mechanisms linking these phenomena require additional study. The relative contributions of direct HA-B cell interactions versus indirect effects mediated through other immune cells or stromal components remain to be fully elucidated [2, 15, 60]. Additionally, the specific receptors and signaling pathways through which different-sized HA fragments influence B-cell function need to be characterized in detail [54]. B cells express several potential HA receptors, including CD44 and RHAMM, which could mediate direct cellular responses to HA [61–63]. High molecular weight HA typically promotes anti-inflammatory responses, while low molecular weight HA fragments can act as DAMPs and may enhance B cell activation [7]. Beyond direct receptor-mediated interactions, HA may influence B cell responses through indirect mechanisms such as modulation of tissue mechanics, antigen presentation, and lymphoid microenvironment organization [18]. Future studies should directly test these hypotheses through several experimental approaches. In vitro B cell activation assays using different molecular weight HA forms could establish direct cause-effect relationships, while selective inhibition of B cell-HA interactions using blocking antibodies against CD44/RHAMM in immunized mice could determine the functional significance of these interactions. Additionally, combination studies in DORmO mice using B cell depletion together with HA synthesis inhibition could determine whether the protective effects of HA are B cell-dependent. Understanding these mechanisms is particularly important for T1D, where targeting HA synthesis or HA-receptor interactions could represent novel therapeutic approaches for modulating B cell-mediated autoimmune responses. The role of HA-binding proteins in modulating these responses also deserves attention. These proteins can significantly alter how HA influences cellular behavior, and their expression patterns might determine tissue-specific responses to HA accumulation [19, 64]. Future studies should examine how these binding partners contribute to the effects we observed and whether they might represent additional therapeutic targets.

Our study also has some limitations that should be acknowledged. While the DORmO mouse model provides a well-characterized system for studying autoimmune responses, the synchronous nature of disease progression in this model may not fully recapitulate the complexity of human autoimmune diseases [25]. Additionally, while 4-MU effectively reduced HA accumulation, its effects on other cellular processes cannot be completely excluded [26]. Our analysis of HA molecular weight distribution, while consistent with previous findings [11], would benefit from more sensitive methodologies such as SEC-MALLS (size exclusion chromatography with multi-angle laser light scattering) or gel electrophoresis to provide more definitive characterization of HA size changes. Alternative approaches such as ultrafiltration could also be employed to quantify the relative amounts of low versus high molecular weight HA species more precisely. Future studies using more selective approaches to manipulate HA synthesis or degradation in specific cell types might help address these limitations.

From a therapeutic perspective, several questions remain to be addressed. The optimal timing and duration of HA synthesis inhibition for treating autoimmune diseases need to be determined [26]. Additionally, the potential benefits of combining HA synthesis inhibition with existing therapies should be explored. It will also be important to investigate whether the effects we observed in lymphoid tissues are similarly present in other autoimmune conditions affecting different organ systems [65].

## 5 | Conclusion

In conclusion, our findings reveal a previously unrecognized accumulation of HA and its spatial association with B cells within lymphoid tissues during autoimmune reactions. The demonstration that pharmacological inhibition of HA synthesis can modulate B cell activation and antibody production suggests new therapeutic strategies for conditions involving dysregulated B cell responses. Future studies building on these observations may lead to more effective treatments that target both immune cells and their tissue microenvironment.

## Figures and Tables

**FIGURE 1 |:**
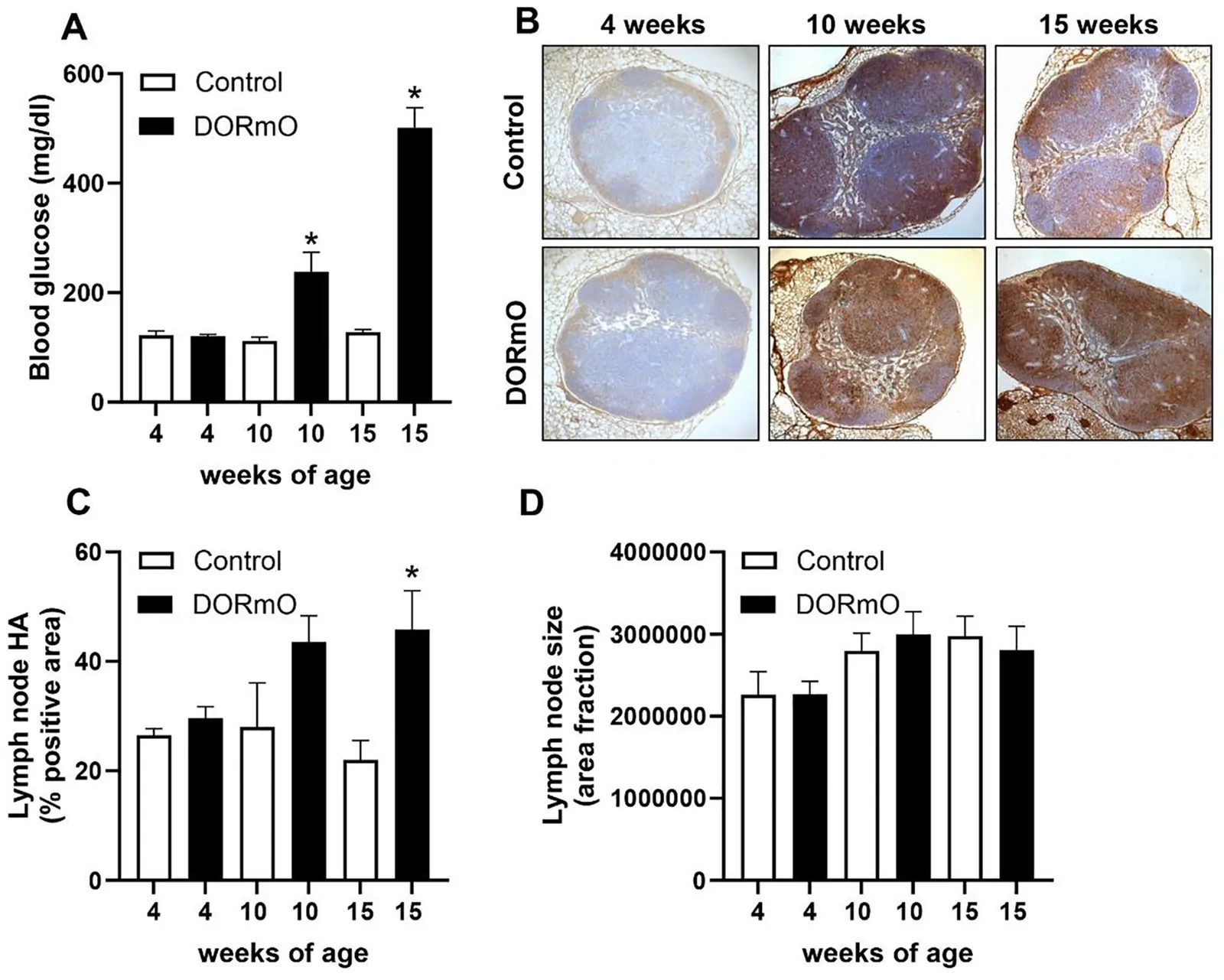
Hyaluronan (HA) deposits accumulate in lymph nodes (LN) in the DORmO mouse model of autoimmune diabetes. (A) The DORmO model is a synchronous model of autoimmune diabetes. Diabetes onset typically occurs at 10 weeks, with all mice diabetic by 15 weeks. (B) Representative histological staining of HA in pancreatic LN tissue from BALB/c (control) and DORmO mice, shown in brown for different time points. (C) Percentage of LN area positive for HA, evaluated from histologic staining of pancreas tissue from control and DORmO mice. (D) Lymph node size. Original magnification, ×40. Bars show mean ± SEM; *p < 0.05 versus control for each time point by unpaired *t*-test. *N* = 6–10 mice per group.

**FIGURE 2 |:**
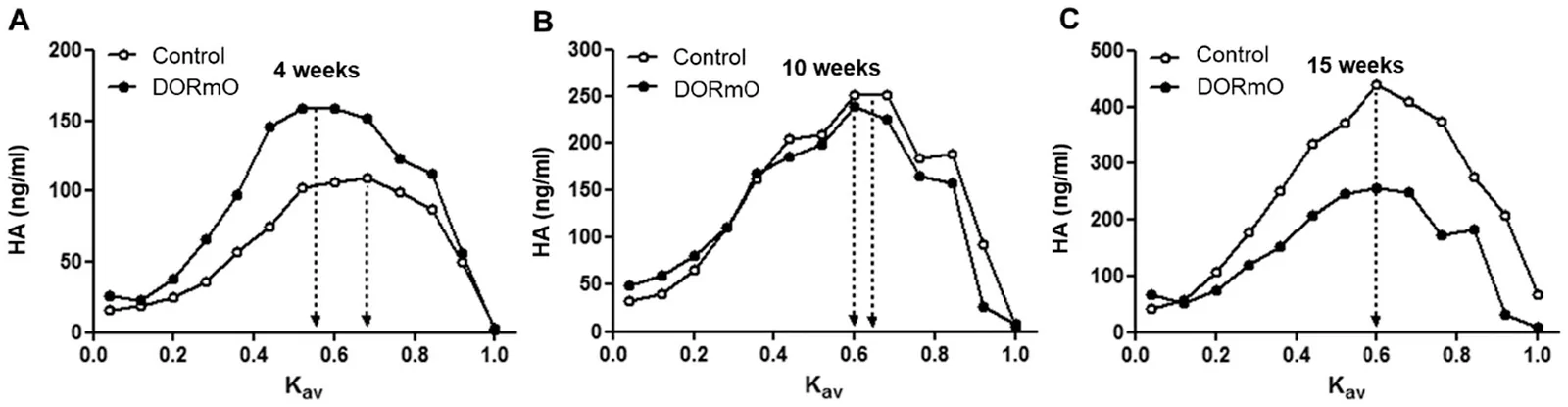
Low molecular mass (LMM)-HA dominates in LN in the DORmO mouse model of autoimmune diabetes. (A) Representative column profile showing HA size distribution from pancreatic LN of 4-week-old BALB/c (control) and DORmO mice. (B) HA size distribution from pancreatic LN of 10-week-old control and DORmO mice. (C) HA size distribution from pancreatic LN of 15-week-old control and DORmO mice. For each time point, HA sizing includes pooled tissues from *N* = 5 mice.

**FIGURE 3 |:**
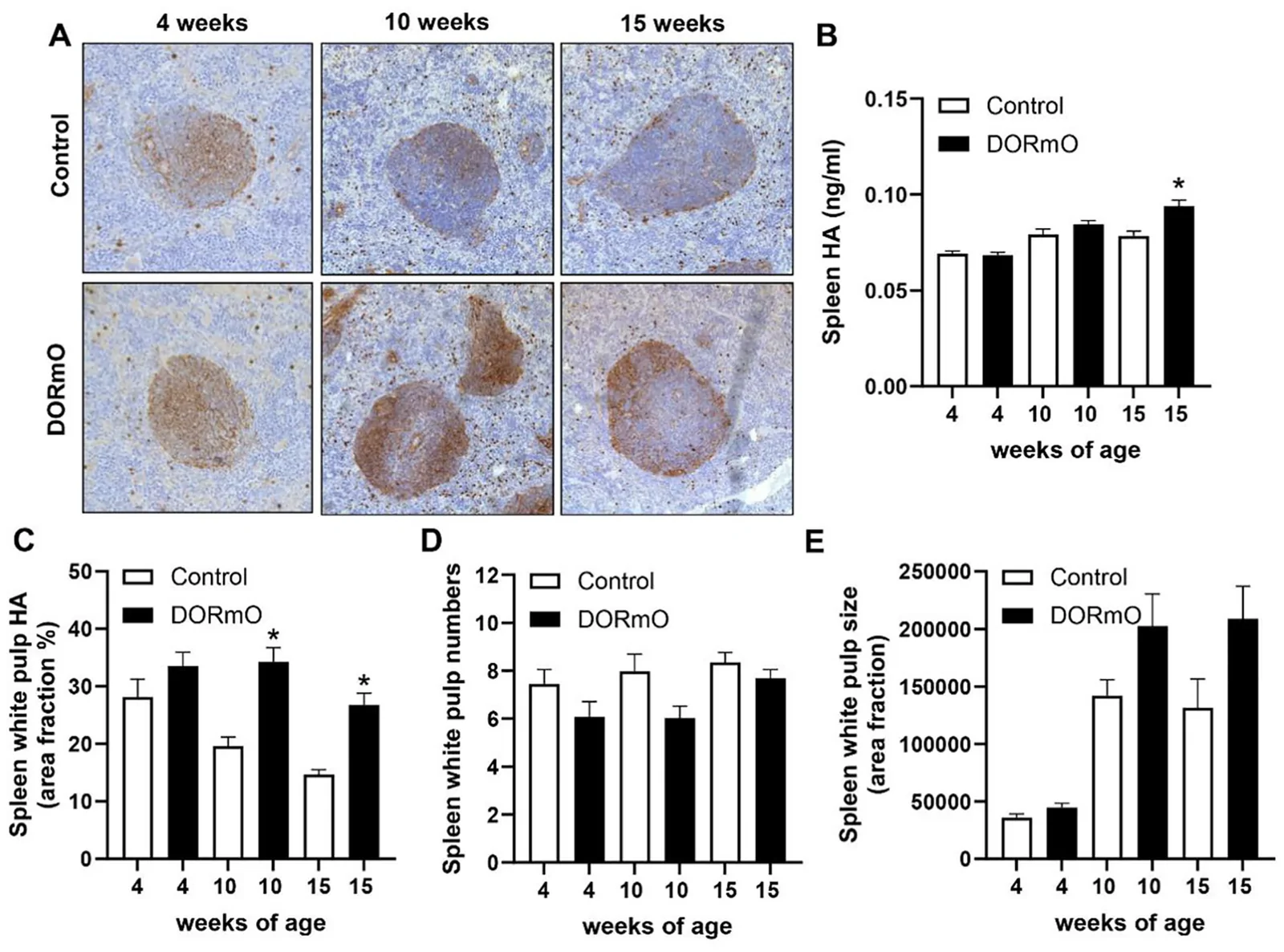
HA deposits accumulate in splenic germinal centers (GC), sites of B-cell activation, in DORmO mice. (A) Representative histological staining of HA in splenic tissue from BALB/c (control) and DORmO mice, shown in brown for different time points. (B) Spleen HA content in control and DORmO mice over time. (C) Average spleen white pulp area positive for HA, evaluated from histologic staining of splenic tissue from control and DORmO mice. (D) Spleen white pulp numbers. (E) Spleen white pulp size. Original magnification, ×20. Bars show mean ± SEM; *p < 0.05 versus control for each time point by unpaired *t*-test. *N* = 5 mice per group.

**FIGURE 4 |:**
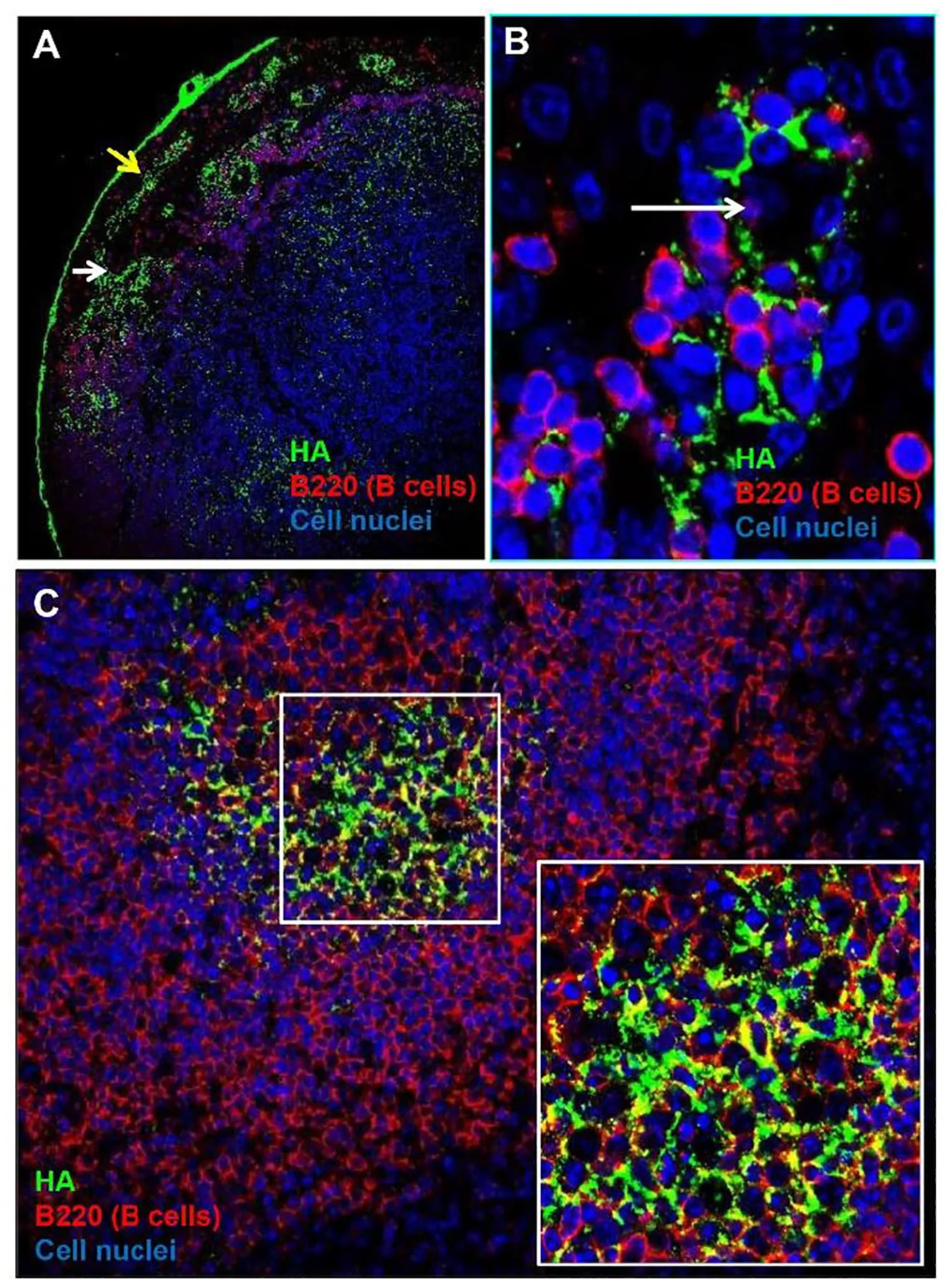
Distribution of HA in mouse LN germinal centers. (A) Localization of HA to the floor of the subcapsular sinus (yellow arrow) and sites of sinus invagination (white arrow). (B) Localization of HA to the lymphatic sinus (white arrow). (C) Abundant HA within LN germinal centers. HA (green), B220, a B cell marker (red), and DAPI, a nucleus dye (blue), are shown.

**FIGURE 5 |:**
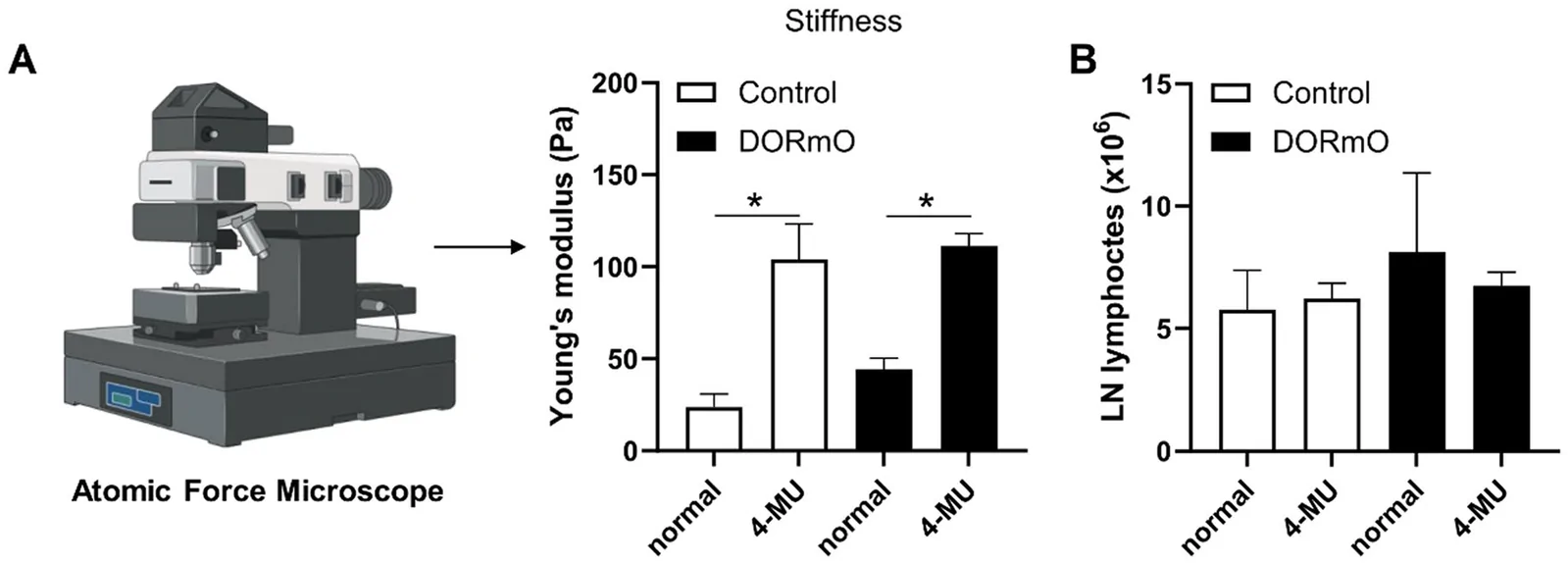
Inhibition of HA synthesis with 4-MU increases LN tissue stiffness. (A) LN stiffnesses. Pancreatic LN were collected from BALB/c (control) and DORmO mice fed either normal chow or 4-MU chow. Stiffness was measured by atomic force microscopy. (B) Totally lymphocyte numbers in LN. Each data point includes pooled tissues from *N* = 5 mice. *p < 0.05 by unpaired *t*-test.

**FIGURE 6 |:**
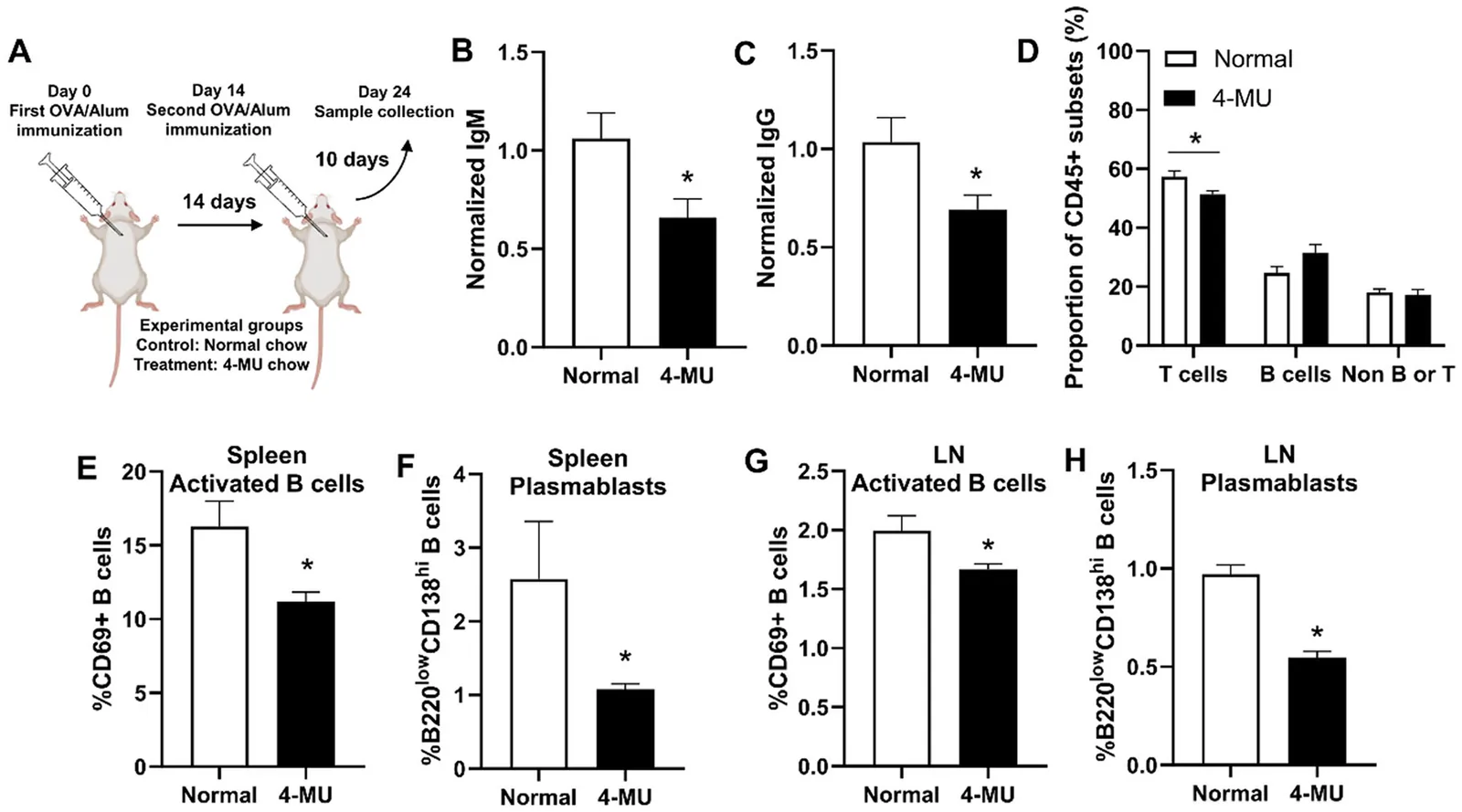
4-MU treatment inhibits antibody production, B-cell activation, and plasmablast formation. (A) Schematic of the experimental design. Mice were immunized with OVA in alum twice, separated by 14 days. Afterwards, serum immunoglobulin analysis and flow cytometry were performed. Mice were initiated on the normal or 4-MU chow at 6 weeks of age and were maintained on the diet until the end of the experiment. (B, C) IgM (B) and IgG (C) levels following immunization in the absence or presence of 4-MU. (D) The proportion of live CD45+ subsets in the spleens of mice on 4-MU or normal chow. (E, F) The percentage of activated B cells (CD19 + CD69+) (E) and plasmablasts (CD19+B220^low^CD138^hi^) (F) in spleens of mice on 4-MU or normal chow. (G, H) The percentage of activated B cells (CD19 + CD69+) (G) and plasmablasts (CD19+B220^low^CD138^hi^) (H) in the LN of mice on 4-MU or normal chow.

## Data Availability

Data will be made available on request.
